# High-quality permanent draft genome sequence of *Ensifer medicae* strain WSM244, a microsymbiont isolated from *Medicago polymorpha* growing in alkaline soil

**DOI:** 10.1186/s40793-015-0119-5

**Published:** 2015-12-10

**Authors:** Julie Ardley, Rui Tian, Graham O’Hara, Rekha Seshadri, T. B. K. Reddy, Amrita Pati, Tanja Woyke, Victor Markowitz, Natalia Ivanova, Nikos Kyrpides, John Howieson, Wayne Reeve

**Affiliations:** Centre for Rhizobium Studies, Murdoch University, Murdoch, Australia; DOE Joint Genome Institute, Walnut Creek, CA USA; Biological Data Management and Technology Center, Lawrence Berkeley National Laboratory, Berkeley, CA USA; Department of Biological Sciences, Faculty of Science, King Abdulaziz University, Jeddah, Saudi Arabia

**Keywords:** Root-nodule bacteria, Nitrogen fixation, Symbiosis, *Alphaproteobacteria*, *Ensifer*, GEBA-RNB

## Abstract

*Ensifer medicae* WSM244 is an aerobic, motile, Gram-negative, non-spore-forming rod that can exist as a soil saprophyte or as a legume microsymbiont of *Medicago* species. WSM244 was isolated in 1979 from a nodule recovered from the roots of the annual *Medicago polymorpha* L. growing in alkaline soil (pH 8.0) in Tel Afer, Iraq. WSM244 is the only acid-sensitive *E. medicae* strain that has been sequenced to date. It is effective at fixing nitrogen with *M. polymorpha* L., as well as with more alkaline-adapted *Medicago* spp. such as *M. littoralis* Loisel., *M. scutellata* (L.) Mill., *M. tornata* (L.) Mill. and *M. truncatula* Gaertn. This strain is also effective with the perennial *M. sativa* L. Here we describe the features of *E. medicae* WSM244, together with genome sequence information and its annotation. The 6,650,282 bp high-quality permanent draft genome is arranged into 91 scaffolds of 91 contigs containing 6,427 protein-coding genes and 68 RNA-only encoding genes, and is one of the rhizobial genomes sequenced as part of the DOE Joint Genome Institute 2010 *Genomic Encyclopedia for Bacteria and Archaea-Root Nodule Bacteria* (GEBA-RNB) project proposal.

**Electronic supplementary material:**

The online version of this article (doi:10.1186/s40793-015-0119-5) contains supplementary material, which is available to authorized users.

## Introduction

Root nodule bacteria that fix atmospheric nitrogen in association with annual and perennial pasture legumes have important roles in agriculture. Some of the most important associations in temperate and Mediterranean regions are the *Ensifer* (*Sinorhizobium*^1^) *- Medicago* symbioses that produce nutritious feed for animals. *Medicago* is a genus within tribe Trifolieae, which is included in the “temperate herbaceous papilionoid” Inverted Repeat Lacking Clade (IRLC) legumes [1, 2]. Species of *Medicago* are amongst the most extensively grown forage and pasture plants and have been cultivated ever since *Medicago sativa* L. (alfalfa, or lucerne) was first domesticated in the Near East and/or Central Asia in about 5000 BC. In addition to perennial *M. sativa* L., annual medic species used widely in agriculture include *M. tornata* (L.) Mill. (disc medic), the model legume *M. truncatula* Gaertn. (barrel medic) and *M. littoralis* Loisel. (strand medic), together with more recently commercialised species such as *M. polymorpha* L. (burr medic) and *M. murex* Willd. (murex medic) [3]. *Medicago* spp. are symbiotically specific: nearly all studied species are nodulated by strains of rhizobia belonging to either *Ensifer medicae* or the closely related species *E. meliloti* [4, 5]. *E. medicae* can be distinguished from *E. melilot*i by its ability to nodulate and fix nitrogen with *M. polymorpha* L. [5].

*Ensifer medicae*WSM244 was isolated in 1979 from a root nodule of *M. polymorpha* L. growing on alkaline soil (pH 8.0) near Tel Afer, Iraq [6]. This strain was superior in N_2_-fixation on a range of medics (*M sativa* L., *M truncatula* Gaertn., *M. tornata* L., *M. polymorpha* L., *M. littoralis* Loisel., *M scutellata* (L.) Mill.) in glasshouse tests in Australia and field trials in Iraq in 1980, and was recommended for development as an inoculant in Iraq (D. Chatel, pers com.). WSM244 has also been used in trials aimed at developing acid-tolerant inoculant strains for pasture medics, as the acid-sensitive nature of the microsymbiont is a constraint to the growth and persistence of *Medicago* spp. in agricultural regions with moderately acidic soils [7]. When field tested in an acidic soil (pH 5.0 CaCl_2_) in Western Australia, WSM244 survived at the site of inoculation for two years, but unlike several more acid tolerant strains it did not demonstrate saprophytic competence and was unable to colonize the soil [8]. This characteristic of WSM244 as an acid-soil sensitive strain correlates with its acid sensitive profile for growth in laboratory media and an inability to maintain a neutral intracellular pH when exposed to pH 6.0 or less [9]. This is in contrast to other *E. medicae* strains, which typically are the dominant microsymbiont partners of annual medics growing on acid soils, in contrast to the more acid-sensitive *E. meliloti*, which preferentially associates with alkaline-soil-adapted *Medicago* spp. [10]. The pH response phenotype of WSM244 is in marked contrast to the sequenced acid tolerant *E. medicae* strain WSM419 [11]. Sequencing the genome of WSM244 and comparing its attributes with an acid-tolerant strain such as WSM419 would provide a means of establishing the molecular determinants required for adaptation to acid soils. This strain was therefore selected as part of the DOE Joint Genome Institute 2010 *Genomic Encyclopedia for Bacteria and Archaea-Root Nodule Bacteria* (GEBA-RNB) sequencing project [12]. Here we present a summary classification and a set of general features for *E. medicae* strain WSM244, together with a description of its genome sequence and annotation.

## Organism information

### Classification and features

**Fig. 1:**
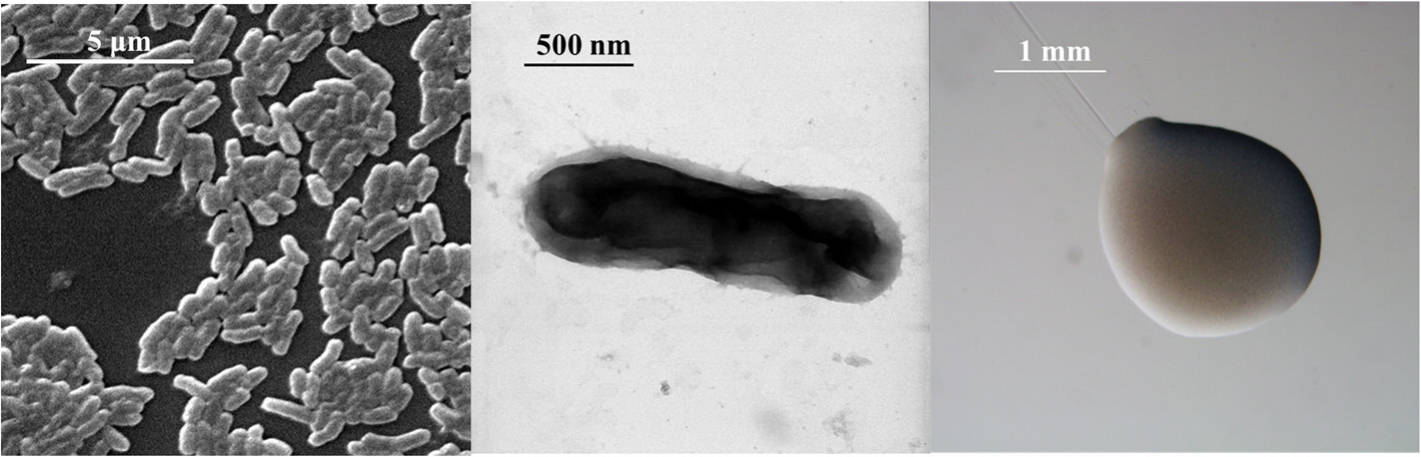
Images of *Ensifer medicae* WSM244 using scanning (*Left*) and transmission (*Center*) electron microscopy and the appearance of colony morphology on solid media (*Right*)

*E. medicae*WSM244 is a motile, Gram-negative rod (Fig. 1 Left and Center) in the order *Rhizobiales* of the class *Alphaproteobacteria*. It is fast growing, forming colonies within 3–4 days when grown on half strength Lupin Agar [13], tryptone-yeast extract agar [14] or a modified yeast-mannitol agar [15] at 28 °C. Colonies on ½LA are white-opaque, slightly domed and moderately mucoid with smooth margins (Fig. 1 Right).

**Fig. 2:**
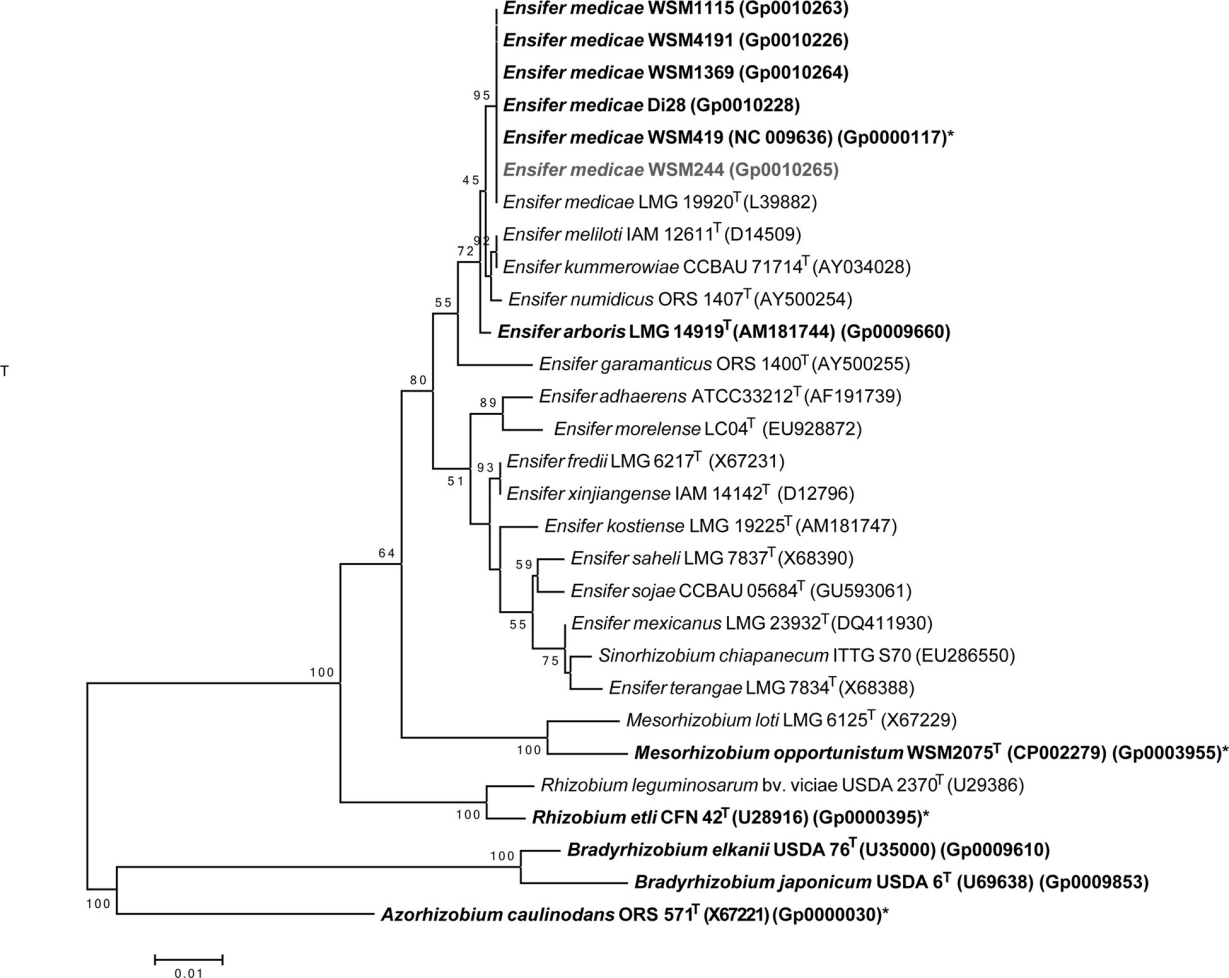
Phylogenetic tree showing the relationship of *Ensifer medicae* WSM244 (shown in bold blue print) to other type and non-type strains in the *Ensifer* genus and to other root nodule bacteria species in the order *Rhizobiales*, based on aligned sequences of the 16S rRNA gene (1,283 bp internal region). (The species name “*Sinorhizobium chiapanecum*” has not been validly published.) *Azorhizobium caulinodans* ORS 571^T^ was used as an outgroup. All sites were informative and there were no gap-containing sites. Phylogenetic analyses were performed using MEGA, version 6 [37]. The tree was built using the Maximum-Likelihood method with the General Time Reversible model [38]. Bootstrap analysis [39] with 500 replicates was performed to assess the support of the clusters. Type strains are indicated with a superscript T. Strains with a genome sequencing project registered in GOLD [17] are in bold font and the GOLD ID is provided after the GenBank accession number. Finished genomes are indicated with an asterisk

Figure 2 shows the phylogenetic relationship of *E. medicae*WSM244 in a 16S rRNA sequence based tree. This strain is the most phylogenetically related to *Ensifer medicae*WSM419 and *Ensifer meliloti*LMG 6133^T^ based on the 16S rRNA gene alignment, with sequence identities of 100 % and 99.71 %, respectively, as determined using the EzTaxon-e database, which contains the sequences of validly published type strains [16]. Minimum Information about the Genome Sequence for WSM244 is provided in Table 1 and Additional file 1: Table S1.

**Table 1:** Classification and general features of *Ensifer medicae* WSM244 in accordance with the MIGS recommendations [40] published by the Genome Standards Consortium [41]

MIGS ID	Property	Term	Evidence code^a^
	Classification	Domain Bacteria	TAS [42]
		Phylum *Proteobacteria*	TAS [43, 44]
		Class *Alphaproteobacteria*	TAS [45, 46]
		Order *Rhizobiales*	TAS [47]
		Family *Rhizobiaceae*	TAS [48]
		Genus *Ensifer*	TAS [49–51]
		Species *Ensifer medicae*	TAS [5]
		Strain: WSM244	TAS [6]
	Gram stain	Negative	IDA
	Cell shape	Rod	IDA
	Motility	Motile	IDA
	Sporulation	Non-sporulating	NAS
	Temperature range	10–40 °C	IDA
	Optimum temperature	25–30 °C	IDA
	pH range; Optimum	6–10; 6.5–8	TAS [9]
	Carbon source	Arabinose, galactose, mannitol, tryptone	TAS [9]
MIGS-6	Habitat	Soil; root nodule on host (*Medicago polymorpha* L.)	TAS [8]
MIGS-6.3	Salinity	0.89–2.0 % (w/v)	NAS
MIGS-22	Oxygen requirement	Aerobic	TAS [8]
MIGS-15	Biotic relationship	Free living, symbiotic	TAS [8]
MIGS-14	Pathogenicity	Biosafety level 1	TAS [52]
MIGS-4	Geographic location	Tel Afer, Iraq	TAS [6]
MIGS-5	Sample collection	1979	TAS [6]
MIGS-4.1	Latitude	36.3833	TAS [6]
MIGS-4.2	Longitude	42.4500	TAS [6]
MIGS-4.3	Depth	0–10 cm	NAS
MIGS-4.4	Altitude	400 m	TAS [6]

^a^Evidence codes—*IDA* Inferred from Direct Assay, *TAS* Traceable Author Statement (i.e., a direct report exists in the literature), *NAS* Non-traceable Author Statement (i.e., not directly observed for the living, isolated sample, but based on a generally accepted property for the species, or anecdotal evidence). These evidence codes are from the Gene Ontology project [53] (http://geneontology.org/page/guide-go-evidence-codes)

#### Symbiotaxonomy

WSM244 nodulates and is effective for nitrogen fixation with *M. littoralis* Loisel., *M sativa* L., *M. tornata* (L.) Mill. [3], *M. murex* Willd., *M. polymorpha* L., *M truncatula* Gaertn. [8] and *M scutellata* (L.) Mill. (D. Chatel per com). WSM244 nodulates and is partially effective for nitrogen fixation with *M. rotata* Boiss. and *M. rugosa* Desr., but does not nodulate *M. blancheana* Boiss. (D. Chatel per com). The symbiotic characteristics of *E. medicae*WSM244 on a range of selected hosts are summarised in Additional file 2: Table S2.

## Genome sequencing information

### Genome project history

This organism was selected for sequencing on the basis of its environmental and agricultural relevance to issues in global carbon cycling, alternative energy production, and biogeochemical importance, and is part of the *Genomic Encyclopedia of Bacteria and Archaea, The Root Nodulating Bacteria* chapter project at the U.S. Department of Energy, Joint Genome Institute. The genome project is deposited in the Genomes OnLine Database [17] and a high-quality permanent draft genome sequence is deposited in IMG [18]. Sequencing, finishing and annotation were performed by the JGI [19]. A summary of the project information is shown in Table 2.

**Table 2:** Genome sequencing project information for *E. medicae* WSM244

MIGS ID	Property	Term
MIGS-31	Finishing quality	High-quality draft
MIGS-28	Libraries used	Illumina Standard shotgun library
MIGS-29	Sequencing platforms	Illumina HiSeq 2000
MIGS-31.2	Fold coverage	677x Illumina
MIGS-30	Assemblers	Velvet version 1.1.04; ALLPATHS v. r41043
MIGS-32	Gene calling methods	Prodigal 1.4
	Locus Tag	A3C7 (http://www.ncbi.nlm.nih.gov/bioproject/?term=A3C7)
	Genbank ID	ATTR00000000
	Genbank Date of Release	July 9 2013
	GOLD ID	Gp0010265 (https://gold.jgi-psf.org/project?id=10265)
	BIOPROJECT	882
MIGS-13	Source Material Identifier	WSM244
	Project relevance	Symbiotic N_2_ fixation, agriculture

### Growth conditions and genomic DNA preparation

*E. medicae*WSM244 was grown on TY solid medium [14] for three days, then a single colony was selected and used to inoculate 5 ml TY broth medium. The culture was grown for 48 h on a gyratory shaker (200 rpm) at 28 °C. Subsequently 1 ml was used to inoculate 60 ml TY broth medium and grown on a gyratory shaker (200 rpm) at 28 °C until OD 0.6 was reached. DNA was isolated from 60 ml of cells using a CTAB bacterial genomic DNA isolation method (http://jgi.doe.gov/collaborate-with-jgi/pmo-overview/protocols-sample-preparation-information/). Final concentration of the DNA was 0.5 mg ml^−1^.

### Genome sequencing and assembly

The draft genome of *E. medicae*WSM244 was generated at the DOE Joint genome Institute (JGI) using the Illumina technology [20]. An Illumina Std shotgun library was constructed and sequenced using the Illumina HiSeq 2000 platform which generated 22,576,268 reads totaling 3,386.4 Mbp. All general aspects of library construction and sequencing performed at the JGI can be found at the JGI website. All raw Illumina sequence data was passed through DUK, a filtering program developed at JGI, which removes known Illumina sequencing and library preparation artifacts ((Mingkun L, Copeland A, Han J. unpublished) . The following steps were then performed for assembly: (1) filtered Illumina reads were assembled using Velvet (version 1.1.04) [21], (2) 1–3 Kbp simulated paired end reads were created from Velvet contigs using wgsim (https://github.com/lh3/wgsim), (3) Illumina reads were assembled with simulated read pairs using Allpaths–LG (version r41043) [22]. Parameters for assembly steps were: 1) Velvet (velveth: 63 –shortPaired and velvetg: −very clean yes –export- Filtered yes –min contig lgth 500 –scaffolding no –cov cutoff 10) 2) wgsim (−e 0 –1 100 –2 100 –r 0 –R 0 –X 0) 3) Allpaths–LG (PrepareAllpathsInputs: PHRED 64 = 1 PLOIDY = 1 FRAG COVERAGE = 125 JUMP COVERAGE = 25 LONG JUMP COV = 50, RunAllpathsLG: THREADS = 8 RUN = std shredpairs TARGETS = standard VAPI WARN ONLY = True OVERWRITE = True) . The final draft assembly contained 91 contigs in 91 scaffolds. The total size of the genome is 6.7 Mbp and the final assembly is based on 789.1 Mbp of Illumina data, which provides an average 118.7x coverage of the genome.

### Genome annotation

Genes were identified using Prodigal [23], as part of the DOE-JGI genome annotation pipeline [24, 25]. The predicted CDSs were translated and used to search the National Center for Biotechnology Information nonredundant database, UniProt, TIGRFam, Pfam, KEGG, COG, and InterPro databases. The tRNAScanSE tool [26] was used to find tRNA genes, whereas ribosomal RNA genes were found by searches against models of the ribosomal RNA genes built from SILVA [27]. Other non–coding RNAs such as the RNA components of the protein secretion complex and the RNase P were identified by searching the genome for the corresponding Rfam profiles using INFERNAL [28]. Additional gene prediction analysis and manual functional annotation was performed within the Integrated Microbial Genomes (IMG) platform [29] developed by the Joint Genome Institute, Walnut Creek, CA, USA [30].

## Genome properties

The genome is 6,650,282 nucleotides with 61.21 % GC content (Table 3) and comprised of 91 scaffolds of 91 contigs. From a total of 6,495 genes, 6,427 were protein encoding and 68 RNA only encoding genes. The majority of protein-coding genes (79.34 %) were assigned a putative function whilst the remaining genes were annotated as hypothetical. The distribution of genes into COGs functional categories is presented in Table 4.

**Table 3:** Genome statistics for *Ensifer medicae* WSM244

Attribute	Value	% of Total
Genome size (bp)	6,650,282	100.00
DNA coding (bp)	5,800,639	87.22
DNA G + C (bp)	4,070,659	61.21
DNA scaffolds	91	100.00
Total genes	6,495	100.00
Protein coding genes	6,427	98.95
RNA genes	68	1.05
Pseudo genes	4	0.06
Genes in internal clusters	2,889	44.48
Genes with function prediction	5,153	79.34
Genes assigned to COGs	4,567	70.32
Genes with Pfam domains	5,311	81.77
Genes with signal peptides	536	8.25
Genes with transmembrane helices	1,467	22.59
CRISPR repeats	0	-

**Table 4:** Number of genes of *Ensifer medicae* WSM244 associated with general COG functional categories

Code	Value	% age of total (4,567)	Description
J	220	4.23	Translation, ribosomal structure and biogenesis
A	0	0.00	RNA processing and modification
K	449	8.62	Transcription
L	120	2.30	Replication, recombination and repair
B	1	0.02	Chromatin structure and dynamics
D	29	0.57	Cell cycle control, cell division, chromosome partitioning
Y	0	0.00	Nuclear structure
V	100	1.92	Defense mechanisms
T	209	4.01	Signal transduction mechanisms
M	265	5.09	Cell wall/membrane/envelope biogenesis
N	71	1.36	Cell motility
Z	0	0.00	Cytoskeleton
W	24	0.46	Extracellular structures
U	68	1.31	Intracellular trafficking, secretion, and vesicular transport
O	187	3.59	Posttranslational modification, protein turnover, chaperones
C	338	6.49	Energy production and conversion
G	574	11.02	Carbohydrate transport and metabolism
E	602	11.56	Amino acid transport and metabolism
F	125	2.40	Nucleotide transport and metabolism
H	225	4.32	Coenzyme transport and metabolism
I	220	4.23	Lipid transport and metabolism
P	286	5.49	Inorganic ion transport and metabolism
Q	160	3.07	Secondary metabolite biosynthesis, transport and catabolism
R	552	10.60	General function prediction only
S	323	6.20	Function unknown
X	47	0.90	Mobilome: prophages, transposons
-	1928	29.68	Not in COGS

## Insights from the genome sequence

WSM244 is one of six strains of *E. medicae* and one of 30 *E. medicae* or *E. meliloti**Medicago*-nodulating strains that have been sequenced and whose genomes have been deposited in the IMG database. The genome of WSM244 falls within the expected size range of 6.4–7.2 Mbp for *E. medicae*. As observed in other *E. medicae* genomes, WSM244 possesses a large number of genes assigned to COG functional categories for: transport and metabolism of amino acids (12.15 %), carbohydrates (11.17 %), inorganic ions (5.3 %), lipids (3.91 %) and coenzymes (3.32 %), transcription (8.63 %) and signal transduction (3.66 %). The WSM244 genome contains only four pseudo genes, the numbers of which are highly variable in sequenced *E. medicae* strains and can be as high as 485 (*E. medicae*WSM4191). All six *E. medicae* strains share high ANI values of 99.18–99.67 %, which is consistent with the low levels of genetic diversity found in *E. medicae* populations [31]. The six *E. medicae* strains share 5,425 core orthologous genes. WSM244 contains 202 unique genes, including those found in clusters encoding a putative polyketide synthase, phage proteins and a sulfonate transport system. Around 72 % of these unique genes encode hypothetical proteins. Strain WSM244 is particularly interesting, as it lacks the acid tolerance of other *E. medicae* strains. The genome of this strain does contain orthologs of acid response or acid tolerance genes that were initially discovered in *E. medicae*WSM419. These genes include *actA* (*lnt*), *actP*, *actR*, *actS*, *phrR*, *lpiA* and *acvB* [32–35]. WSM244 also contains the *tcsA*-*tcrA*-*fsrR*- regulatory gene cluster which is required for the low-pH-activation of *lpiA* and *acvB* in *E. medicae*WSM419 [36]. This finding is in direct contrast to the absence of *fsrR*, *tcsA* and *tcrA* in the the acid-sensitive strain *E. meliloti* 1021. This suggests that either there may be differences in pH responsive gene expression in the WSM244 background, or that acid tolerant *E. medicae* strains possess other candidate genes that are required for low pH adaptation and have not yet been identified.

## Conclusions

WSM244 is of particular interest as it was isolated from *M. polymorpha* growing in alkaline soil and it lacks the acid tolerance of *E. medicae* strains isolated from medics growing in acid Sardinian and Greek soils [9]. WSM244 is the only acid-sensitive *E. medicae* strain that has been sequenced to date. Analysis of its sequenced genome and comparison with other sequenced *E. medicae* and *E. meliloti* genomes will yield new insights into the molecular basis of acid tolerance in rhizobia and into the ecology and biogeography of the *Ensifer-Medicago* symbiosis.

## Additional files

Additional file 1: Table S1. Associated MIGS record for WSM244. (DOCX 19 kb) Additional file 2: Table S2. Nodulation and N_2_ fixation properties of *E. medicae* WSM244 on selected *Medicago* spp. Data compiled from [3, 6, 8]. Note that ‘+’ and ‘-’ denote presence or absence, respectively, of nodulation (Nod) or N_2_ fixation (Fix). (DOCX 15 kb)

## Abbreviations

GEBA-RNB: Genomic Encyclopedia for Bacteria and Archaea-Root Nodule Bacteria
IRLC: Inverted Repeat Lacking Clade

## Footnotes

1 *Editorial note*—Readers are advised that in Opinion 84 the Judicial Commission of the International Committee on Systematics of Prokaryotes ruled that the genus name *Ensifer* Casida 1982 has priority over *Sinorhizobium* Chen et al. 1988 and the names are synonyms [1]. It was further concluded that the transfer of members of the genus *Sinorhizobium* to the genus *Ensifer,* as proposed by Young [2] would not cause confusion.
