# Supplementary material for: High-quality permanent draft genome sequence of Ensifer medicae strain WSM244, a microsymbiont isolated from Medicago polymorpha growing in alkaline soil
Source: Stand Genomic Sci. 2015 Dec 10;10:126. doi: 10.1186/s40793-015-0119-5 (PMC4674904; doi:10.1186/s40793-015-0119-5)
Supplement: Additional file 2: Table S2. — Nodulation and N2 fixation properties of E. medicae WSM244 on selected Medicago spp. Data compiled from [3, 6, 8]. Note that ‘+’ and ‘-’ denote presence or absence, respectively, of nodulation (Nod) or N2 fixation (Fix). (DOCX 15 kb) [file 40793_2015_119_MOESM2_ESM.docx]

**Table S2**. Nodulation and N_2_ fixation properties of [*E. medicae*](http://dx.doi.org/10.1601/nm.1334) [WSM244](http://doi.org/10.1601/strainfinder?urlappend=%3Fid%3DWSM244) on selected *Medicago* spp. Data compiled from [3, 6, 8]. Note that ‘+’ and ‘-’ denote presence or absence, respectively, of nodulation (Nod) or N_2_ fixation (Fix).

| **Species Name** | **Cultivar or Accession** | **Growth Habit** | **Nodulation** | **N_2_ fixation** | **Comment** |
| --- | --- | --- | --- | --- | --- |
| *M. blancheana* Boiss. |  | Annual | Nod^-^ | Fix^-^ | No nodulation |
| *M. littoralis* Loisel. | Harbinger | Annual | Nod^+^ | Fix^+^ | Effective |
| *M. murex* Willd. |  | Annual | Nod^+^ | Fix^+^ | Effective |
| *M. polymorpha* L. | Serena | Annual | Nod^+^ | Fix^+^ | Highly effective |
| *M. rigidula* (L.) All. |  | Annual | Nod^+^ | Fix^+^ | Highly effective |
| *M. rotata* Boiss. |  | Annual | Nod^+^ | Fix^+^ | Poorly effective |
| *M. rugosa* Desr. |  | Annual | Nod^+^ | Fix^+^ | Poorly effective |
| *M. sativa* L. |  | Perennial | Nod^+^ | Fix^+^ | Highly effective |
| *M. scutellata* (L.) Mill. |  | Annual | Nod^+^ | Fix^+^ | Highly effective |
| *M. tornata* (L.) Mill. | Swan | Annual | Nod^+^ | Fix^+^ | Effective |
| *M. tornata* (L.) Mill. | Tornafield | Annual | Nod^+^ | Fix^+^ | Effective |
| *M. truncatula* Gaertn. | Cyprus | Annual | Nod^+^ | Fix^+^ | Highly effective |
